# Pseudohypoaldosteronism in a Neonate Presenting as Life-Threatening Hyperkalemia

**DOI:** 10.1155/2016/6384697

**Published:** 2016-01-19

**Authors:** Najya A. Attia, Yousef I. Marzouk

**Affiliations:** ^1^Department of Pediatric Endocrinology and Metabolism, King Abdulaziz Medical City, Jeddah 21423, Saudi Arabia; ^2^King Saud bin Abdulaziz University for Health Sciences, Jeddah 21423, Saudi Arabia

## Abstract

*Context*. Pseudohypoaldosteronism type 1 (PHA1) is a life-threatening disease that causes severe hyperkalemia and cardiac arrest if not treated appropriately or if diagnosis is missed.* Objective.* To report a case of a newborn with vomiting and lethargy, ultimately diagnosed with pseudohypoaldosteronism.* Patient.* This case presented to the ED at an age of 14 days in hypovolemic shock. There was a family history of sudden infant death, her sister who was diagnosed with CAH and passed away at 3 months of age despite regular hormone replacement. Our patient had cardiac arrest in ED, due to hyperkalemia; while receiving fluid boluses, cardiopulmonary resuscitation was initiated. After stabilization, diagnostic workup demonstrated persistently low sodium, acidosis, and high potassium, which required peritoneal dialysis. Based on these findings, the patient was diagnosed with CAH. It turned out later that the patient had PHA1. Two years later, the patient had a new sibling with the same disease diagnosed at birth and started immediately on treatment without any complication.* Conclusions and Outcome.* This case highlights the significant diagnostic and therapeutic challenges in treating children with PHA1. Adrenal crisis is not always CAH; delayed diagnosis can lead to complication and even death. The presence of high plasma renin activity, aldosterone, and cortisol, along with the presence of hyponatremia and hyperkalemia, established the diagnosis of PHA type 1 and ruled out CAH.

## 1. Background

Pseudohypoaldosteronism (PHA) type 1 is a rare hereditary disorder caused by resistance to the aldosterone action. It is characterized by hyperkalemia, hyponatremia, metabolic acidosis, and high plasma aldosterone and renin concentrations [1]. PHA1 is further classified into renal (AD) and systemic (AR) [2]. Renal PHA1 is an autosomal dominant (AD) disorder with heterogeneous mutations on the gene coding for the aldosterone receptor [3], in which salt loss is restricted to the kidney. In contrast, systemic PHA1 has an autosomal recessive (AR) pattern of inheritance caused by loss-of-function mutation of the epithelial sodium channel (ENaC) [4, 5]; it is characterized by generalized salt loss in many organs, including the lung, kidney, colon, and sweat and salivary glands [6, 7].

PHA1 usually presents in the neonatal period with hypovolemia, hyponatremia, hyperkalemia, and metabolic acidosis [8–11]. Its generalized presentation and lack of specific clinical features, in addition to the rarity of the disease, usually results in delayed diagnosis and an increased chance of mortality. Salt-wasting forms of congenital adrenal hyperplasia (CAH) are more common than PHA1 but have a similar presentation, such that health providers think of CAH first and start the patient on hydrocortisone and fludrocortisone, which are not effective in managing PHA1. Such incorrect management not only delays diagnosis but also complicates the case and can increase mortality.

We present the case of a 14-day-old baby girl who presented to the emergency department (ED) with life-threatening hyperkalemia. She was treated for CAH but did not respond to treatment, and it was found that the hyperkalemia was due to PHA1 rather than CAH. The patient ultimately required peritoneal dialysis to control her hyperkalemia.

## 2. Clinical Case

The patient presented to the ED at the age of 14 days in hypovolemic shock. She was the second child of consanguineous parents and had a birth weight of 2.9 kg.

There was a family history of sudden infant death. A female sibling died at 3 months of age after being diagnosed with CAH and receiving appropriate glucocorticoid and mineralocorticoid replacement.

Our patient had cardiac arrest in the ED while receiving fluid boluses; the cardiac monitor showed peaked T waves and wide QRS complex, indicating hyperkalemia. Cardiopulmonary resuscitation was initiated, intubation was performed, and the patient was immediately started on hyperkalemia management, including intravenous calcium gluconate, nebulized salbutamol, insulin, and glucose infusion, intravenous sodium bicarbonate, and oral calcium resonium (potassium binding resin).

Initial laboratory findings showed hyperkalemia (8.61 mmol/L), hyponatremia (122 mmol/L), and acidosis (arterial pH = 6.9; serum bicarbonate = 4.7 mmol/L). Intravenous sodium bicarbonate was given to improve acidosis and hyperkalemia; after three courses of nebulized salbutamol, insulin, and glucose infusion, the potassium level began to decline (6.73 mmol/L) and the patient was admitted to the pediatric intensive care unit (PICU).

We considered adrenal crisis due to CAH in view of the patient's hyperkalemia, hyponatremia, acidosis, and family history of CAH with consanguineous parents. Blood was extracted for cortisol, adrenocorticotropic hormone (ACTH), renin, and aldosterone; then, the patient was started on hydrocortisone and fludrocortisone as a suspected case of CAH. The patient was not responding to treatment; her potassium fell to 6.52 mmol/L before rising again to 9.24 mmol/L and becoming refractory. The patient had cardiac arrest again and was resuscitated. Emergency peritoneal dialysis was initiated, after which the potassium level began to decline.  Table 1 summarizes laboratory findings before and after initiation of peritoneal dialysis.  Table 2 shows the results of the hormonal studies. These findings ruled out CAH as the cause for the infant's physiologic collapse and hyperkalemia. The diagnosis of PHA type 1 was assigned based on the elevated renin and aldosterone levels.

Hydrocortisone and fludrocortisone were discontinued. The patient initially required parenteral sodium chloride and sodium bicarbonate, but subsequently oral sodium chloride and sodium bicarbonate were initiated. Oral calcium resonium was continued at a dosage of about 1-2 g/kg/day, and the potassium level was kept under check with adjustment to dosage according to the potassium level. The baby was discharged after her potassium and sodium levels and acidosis were corrected and found to be stable.

Following her initial presentation, the child developed a skin rash diagnosed as eczema. She was admitted once when she developed gastroenteritis with severe dehydration. Management included intravenous sodium chloride, sodium bicarbonate, and treatment of hyperkalemia. Outpatient treatment included oral sodium chloride, sodium bicarbonate, and calcium resonium. The child is now 7 years old and has normal growth and development. A female sibling born when our patient was 3 years old had biochemical evidence of PHA type 1. Therapy was initiated to prevent a similar crisis.

## 3. Discussion

Aldosterone is a steroid hormone that regulates sodium and potassium homeostasis; it acts primarily on epithelial cells in the renal collecting ducts [8]. Aldosterone crosses the epithelial cell membrane and binds to the mineralocorticoid receptor (MR), which acts as a ligand-dependent transcription factor in target tissues, thereby promoting gene signaling [9, 10]. Transcription of signaling factors enhances sodium transport into the epithelial cell, which is then actively removed from the cell by the sodium-potassium ATPase on the basolateral membrane of the cell [11]. Inactivating mutations of the gene encoding the aldosterone receptor cause renal PHA type 1, while systemic PHA type 1 is caused by inactivating mutations of the ENaC subunit genes [4, 5].

The clinical spectrum of renal PHA varies widely from asymptomatic patients diagnosed only with elevated renin and aldosterone to patients presenting with a salt-wasting crisis. More than 50 different mutations have been described but there are no genotype/phenotype correlations. The generalized form of PHA type 1 leads to salt loss from organs expressing ENaC, including the kidneys, salivary and sweat glands, and the colon. Patients typically present shortly after birth with electrolyte findings mimicking adrenal crisis. It is usually severe and persists into adulthood.

The electrolyte abnormalities of hyponatremia, hyperkalemia, and metabolic acidosis are similar in CAH and PHA. However, in contrast to CAH, serum cortisol and aldosterone levels are elevated in PHA indicating normal glucocorticoid production and resistance to aldosterone activity. The patient presented here clinically fits the picture of systemic PHA1 with severe refractory hyperkalemia requiring peritoneal dialysis at diagnosis and continued requirement for salt supplementation. Systemic PHA1 is also associated with other clinical features including skin changes [9], increased risk of respiratory infections [7], polyhydramnios [12], and cholelithiasis [13]. Our patient has persistent skin disease and continues to require salt supplementation calcium resonium to manage hyperkalemia. Genetic analysis to confirm an ENaC mutation was not feasible.

Hyperkalemia is a life-threating condition [11]; it requires early recognition and aggressive treatment. Both CAH and PHA1 present in the neonatal stage with a similar clinical picture (weight loss, poor feeding, dehydration, and lethargy) and laboratory results suggestive of adrenal crisis (hyperkalemia, hyponatremia, and acidosis). Treatment with hydrocortisone should be considered in all patients with no index case of PHA, especially patients with ambiguous genitalia or male patients, because hydrocortisone results in an excellent response in the case of CAH. However, it is very important to send blood for 17-hydroxyprogesterone (17-OHP) aldosterone, renin, cortisol, and ACTH before starting hydrocortisone. When the patient is female with normal genitalia or the response to corticosteroids is poor, resistance to aldosterone should always be considered.

Treatment of PHA1 should be considered as an emergency and life-saving measure. This comprises adequate rehydration, replacement of salt loss, and correction of hyperkalemia and acidosis in the acute phase.

After initial stabilization, potassium exchange resins and salt supplementation are the mainstays of treatment. The indomethacin and thiazide diuretics have been tried but with limited and variable success rates.

## Figures and Tables

**Table 1:** Effect of peritoneal dialysis on blood chemistry.

Investigation	Before peritoneal dialysis	After peritoneal dialysis
Serum sodium	122 mmol/L	134 mmol/L (135–144)
Serum chloride	95 mmol/L	92 mmol/L (101–111)
Serum potassium	9.24 mmol/L	6.42 mmol/L (3.5–4.9)
Serum bicarbonate	11 mmol/L	25 mmol/L (23–31)
Blood urea	7.1 mmol/L	6.4 mmol/L (1.9–5.7)
Serum creatinine	64 umol/L	71 umol/L (50–74)

**Table 2:** Results of hormonal studies.

Investigation	Results (normal range)
Serum renin activity	28.6 ng/mL/h (0.1–3.1)
Serum aldosterone	145 ng/dL (3–16)
Serum cortisol	87.9 ug/dL (2.3–11.9)

## Abbreviations

PHA1: Pseudohypoaldosteronism type 1
CAH: Congenital adrenal hyperplasia
ED: Emergency department
MTOD: Multitarget organ defect
ENaC: Epithelial sodium channel.
